# Improved skeletal muscle fatigue resistance in experimental autoimmune myositis mice following high-intensity interval training

**DOI:** 10.1186/s13075-022-02846-2

**Published:** 2022-06-27

**Authors:** Takashi Yamada, Yuki Ashida, Katsuyuki Tamai, Iori Kimura, Nao Yamauchi, Azuma Naito, Nao Tokuda, Håkan Westerblad, Daniel C. Andersson, Koichi Himori

**Affiliations:** 1 Graduate School of Health Sciences, Sapporo Medical University, Sapporo, 060-8556 Japan; 2 Research fellow of Japan Society for Promotion of Science, Tokyo, Japan; 3 Department of Physiology and Pharmacology, Karolinska Institutet, Stockholm, Sweden; 4 Heart, Vascular and Neurology Theme, Cardiology Unit, Karolinska University Hospital, Stockholm, Sweden

## Abstract

**Background:**

Muscle weakness and decreased fatigue resistance are key manifestations of systemic autoimmune myopathies (SAMs). We here examined whether high-intensity interval training (HIIT) improves fatigue resistance in the skeletal muscle of experimental autoimmune myositis (EAM) mice, a widely used animal model for SAM.

**Methods:**

Female BALB/c mice were randomly assigned to control (CNT) or EAM groups (*n* = 28 in each group). EAM was induced by immunization with three injections of myosin emulsified in complete Freund’s adjuvant. The plantar flexor (PF) muscles of mice with EAM were exposed to either an acute bout or 4 weeks of HIIT (a total of 14 sessions).

**Results:**

The fatigue resistance of PF muscles was lower in the EAM than in the CNT group (*P* < 0.05). These changes were associated with decreased activities of citrate synthase and cytochrome c oxidase and increased expression levels of the endoplasmic reticulum stress proteins (glucose-regulated protein 78 and 94, and PKR-like ER kinase) (*P* < 0.05). HIIT restored all these alterations and increased the peroxisome proliferator-activated receptor γ coactivator-1α (PGC-1α) and the mitochondrial electron transport chain complexes (I, III, and IV) in the muscles of EAM mice (*P* < 0.05).

**Conclusions:**

HIIT improves fatigue resistance in a SAM mouse model, and this can be explained by the restoration of mitochondria oxidative capacity via inhibition of the ER stress pathway and PGC-1α-mediated mitochondrial biogenesis.

## Background

Patients with systemic autoimmune myopathies (SAMs), including polymyositis (PM), dermatomyositis (DM), and juvenile DM, suffer from muscle weakness and reduced fatigue resistance [1], which are associated with debility and increased mortality [2, 3]. The decreased endurance exercise capacity in patients with PM/DM is accompanied by a lower maximal oxygen uptake (VO_2_ max) [2, 4]. Moreover, mitochondrial dysfunction has been observed in the skeletal muscle of PM/DM patients [2, 5, 6], suggesting a low mitochondrial oxidative capacity as an important mechanism contributing to the reduced fatigue resistance in these patients.

The mechanisms that lead to impaired mitochondrial function in SAMs are not fully clarified. Although mitochondrial dysfunction has been shown to be associated with a proinflammatory microenvironment [7], there is growing evidence showing that other factors are also involved [8]. In this regard, the endoplasmic reticulum (ER) stress pathways are chronically activated in SAMs [9] and are associated with impaired mitochondrial function [10, 11]. A previous study has suggested a potential mechanistic link between sustained ER stress and mitochondrial dysfunction, mediated by reactive oxygen/nitrogen species generation due to augmented Ca^2+^ transfer through the mitochondrial-associated ER membrane (MAMs) [8, 11, 12].

Over a period of years, physical exercise was not recommended to patients with SAMs due to fear of exacerbating muscle inflammation. However, since the safety and benefits of exercise in SAM patients were first shown in 1993 [13, 14], exercise training emerged as a non-pharmacological therapy to improve muscle function and prevent disease progression [15–17]. For instance, endurance training improved VO_2_ max and mitochondrial enzyme activities with reduced disease activity and downregulation of genes related to ER stress in PM/DM patients [2, 18]. Following a resistance exercise training program, improved muscle strength and increased VO_2_ max were seen in patients with PM/DM, and these improvements were accompanied by a reduction in the gene expression associated with inflammation and fibrosis [19].

A growing body of evidence demonstrates that high-intensity interval training (HIIT) can serve as an effective alternate to traditional endurance training in healthy individuals and diseased populations [20]. Improvements in aerobic capacity are linked to enhanced peripheral oxygen extraction by the skeletal muscle especially after a few weeks of HIIT [21, 22]. By using in vivo neuromuscular electrical stimulation, we recently demonstrated that the HIIT-induced increase in fatigue resistance is larger with high-intensity than with low-intensity contractions in mouse skeletal muscle, and this effect was linked to improved mitochondria content and function [23].

Peroxisome proliferator-activated receptor γ coactivator-1α (PGC-1α) is regarded as an important regulator of mitochondrial biogenesis and function [24]. The AMP-activated protein kinase (AMPK), acetyl-CoA carboxylase (ACC), Ca^2+^/calmodulin-dependent protein kinase II (CaMKII), and p38 mitogen-activated protein kinase are well-known modulators of PGC-1α expression in the skeletal muscle [24–27]. Previous studies have demonstrated that HIIT increases the phosphorylation levels of these signaling molecules and hence increases the expression of PGC-1α [23, 28]. Moreover, activation of AMPK has been shown to inhibit ER stress and inflammation in the skeletal muscle [29].

One of the widely used animal models for SAMs is the experimental autoimmune myositis (EAM) mouse [30]. EAM is induced by immunization with three injections of myosin emulsified in complete Freund’s adjuvant. Muscle function is impaired at the end of the immunization period where muscular inflammation is already established [31, 32]. Intriguingly, we recently have reported that resistance training starting 1 day after the last immunization inhibits ER stress and restores muscle strength in mice with EAM [31]. In the present study, we tested the following hypotheses: (1) fatigue resistance is decreased in the muscle of mice with EAM due to the decreased mitochondrial oxidative capacity induced by ER stress and (2) HIIT combats these deleterious effects of EAM.

## Methods

### Ethical approval

All experimental procedures were approved by the Committee on Animal Experiments of Sapporo Medical University (No. 18-030). Animal care was in accordance with institutional guidelines.

### Induction of experimental autoimmune myositis

Female BALB/c mice (8 weeks old, *n* = 28) and male Wistar rat (9 weeks old, *n* = 1) were supplied by Sankyo Lab Service (Sapporo, Japan). Mice were given food and water ad libitum and housed in an environmentally controlled room (24 ± 2 °C) with a 12-h light-dark cycle. Health was monitored by weight and general assessment of animal activity (every other day). EAM was induced by immunizing mice with partially purified myosin, including myosin-binding protein C, as reported previously [30, 33]. Briefly, the skeletal muscle (30 g) obtained from a Wistar rat was minced and washed four times in 30 mM KCl/150 mM sodium phosphate buffer (pH 7.5), 1 mM EDTA, and 1 mM DTT. Myosin was extracted by incubation of the muscle sample with 90 ml chilled 300 mM KCl/150 mM phosphate buffer containing 5 mM MgCl_2_, 5 mM ATP, 1 mM DTT, and 1 mM EDTA on ice for 45 min with constant agitation. The homogenate was centrifuged for 30 min at 4 °C at 2200*g*. For myosin precipitation, the supernatant was collected, filtered, and diluted with 15 volumes of chilled ultrapure water. The precipitate was recovered via centrifugation for 10 min at 4 °C at 10,000*g*, dissolved in 500 mM KCl, and stored at – 80 °C. Purified rat myosin (10 mg/ml) was emulsified with an equal amount of complete Freund’s adjuvant (Difco) with 3.3 mg/ml *Mycobacterium butyricum* (Difco). BALB/c mice were each immunized intracutaneously with 50–100 μl of an emulsion into three to four locations (a total of 200 μl) on the back on days 0, 7, and 14. One hour after the first immunization, pertussis toxin (500 ng in 100 μl saline; List Biological Laboratories) was intraperitoneally injected into each animal. In the present study, all treated animals underwent successful EAM, defined by a significant increase in spleen weight.

### Experimental design

To assess the molecular and physiological adaptations induced by HIIT in the skeletal muscle of EAM mice, we performed two separate experiments. The primary outcome of this study will be fatigue resistance. Secondary outcomes constitute mitochondrial enzyme activity, the amount of mitochondrial respiratory complexes and ER stress-related proteins, myosin heavy chain (MyHC) isoforms, and the phosphorylation levels of signaling proteins.

#### Experiment 1

**Fig. 1:**
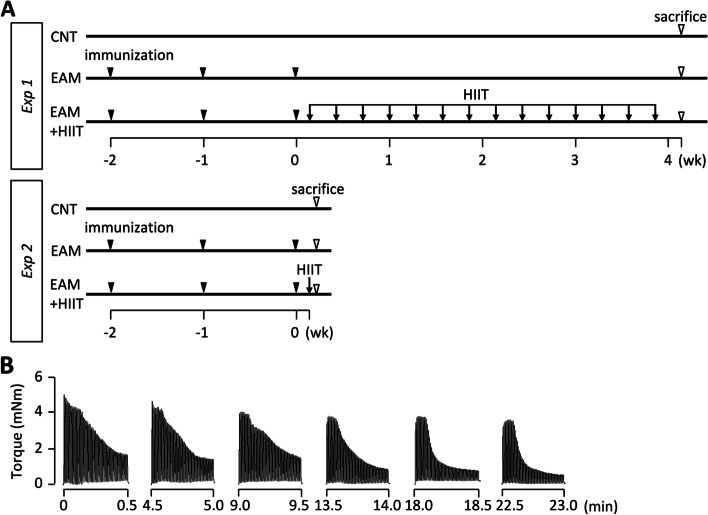
Schematic overview of the experimental design. **A** In experiment (*Exp*) *1*, the fatigue resistance and intracellular events were evaluated under control conditions (CNT) and after induction of experimental autoimmune myosistis (EAM) with and without subsequent high-intensity interval training (HIIT). HIIT was initiated the day after the last immunization to induce EAM and was performed with electrical stimulation of the left leg (EAM + HIIT) every other day for a total of 14 sessions. The right leg served as a non-training EAM control. In *Exp 2*, cellular signaling that underlies the HIIT-induced physiological adaptations was investigated after an acute single bout of HIIT. **B** Typical torque traces of a HIIT session

We first examined the effect of HIIT on muscle fatigability and ER/mitochondrial adaptation in EAM mice. Female BALB/c mice (*n* = 12) were randomly assigned to CNT (*n* = 6) and EAM (*n* = 6) groups. Random numbers were generated using the standard = RAND() function in Microsoft Excel. In the EAM group, HIIT was performed on the left leg (referred to as the EAM + HIIT group), and the right leg served as a non-training EAM control. HIIT was started 24 h after the last immunization and was carried out every other day for a total of 14 sessions (Fig. 1A). The training order was randomized daily, with each animal trained at a different time each training day. Under isoflurane anesthesia, mice were placed supine on a platform with the foot secured to a footplate connected to a torque sensor (S-14154, Takei Scientific Instruments) at an angle of 0° dorsiflexion (i.e., 90° relative to the tibia). The plantar flexor muscles were activated by supramaximal (45 V, 0.5 ms) monophasic rectangular current pulses via a pair of surface electrodes. The stimulation scheme was designed to mimic the activation pattern during all-out cycling bouts, i.e., 0.25 s contractions produced every 0.5 s [23, 34]. Each session consisted of six sets of 60 contractions at 4-min intervals. Twenty-four hours after the last HIIT session, in vivo fatigue resistance of the plantar flexor muscles in each group was measured by 80 repeated 350 ms, 70 Hz tetani given at an interval of 3 s. This was done by an investigator unaware of the treatment side. Twenty-four hours after the measurement of fatigue resistance (i.e., 48 h after the last HIIT session), mice were killed by cervical dislocation under isoflurane anesthesia and the gastrocnemius (GAS) and the plantaris muscles were used for skinned muscle fiber experiments and for biochemical analyses (see below).

#### Experiment 2

To investigate cellular signaling that underlies the HIIT-induced physiological adaptations, female BALB/c mice (*n* = 16) were randomly divided into the same groups as in *experiment 1* (*n* = 8 in each group). Immediately after one HIIT session, mice were killed by rapid cervical dislocation under isoflurane anesthesia, and the muscles were subsequently isolated. The phosphorylation levels of AMPKα Thr172, CaMKII Thr286, ACC Ser79, and p38 MAPK The180/Tyr182 were investigated in the GAS muscles of each animal.

### Myosin heavy chain isoforms separation

Aliquots of GAS muscle extracts containing 5 μg protein were used for myosin heavy chain (MyHC) electrophoresis as previously described [35]. Using a 6.8% polyacrylamide slab gel, electrophoresis was run at 4 °C for 24 h at 160 V and stained with Coomassie brilliant blue. Images of gels were densitometrically evaluated with ImageJ.

### Measurement of Ca^2+^-activated force in skinned muscle fibers

Chemically skinned muscle fibers were prepared, and Ca^2+^-activated force was measured as described previously [36]. The GAS muscle was pinned out at resting length under paraffin oil and kept at 4 °C. Single muscle fibers were dissected under a stereomicroscope. Four to six skinned fibers were obtained from one whole muscle. A segment of the skinned fiber was connected to a force transducer (Muscle Tester, World Precision Instruments) and then incubated with a *N*-2-hydroxyethylpiperazine-*N*′-2-ethanesulfonic acid (HEPES)-buffered solution (see below) containing 1% (vol/vol) Triton X-100 for 10 min in order to remove the membranous structures. Fiber length was adjusted to optimal length (2.5 μm) by laser diffraction as described previously [37], and the contractile properties were measured at room temperature (24 °C).

All solutions were prepared as described in detail elsewhere [38]. They contained (in mM) 36 Na^+^, 126 K^+^, 90 HEPES, 8 ATP, and 10 creatine phosphate and had a pH of 7.09–7.11 and a free Mg^2+^ concentration set at 1.0 mM. The maximum Ca^2+^ solution contained 49.5 mM Ca-EGTA and 0.5 mM free EGTA, whereas the relaxation solution contained 50 mM free EGTA. Various pCa (-log free Ca^2+^ concentration) solutions (pCa 6.4, 6.2, 6.0, 5.8, 5.6, 5.4, and 4.7) were prepared by mixing the maximum Ca^2+^ solution and the relaxation solution in appropriate proportions [39]. The contractile apparatus was directly activated by exposing the skinned fiber to the various pCa solutions, and force was measured. The isometric force produced at each pCa was expressed as a percentage of the corresponding maximum force and analyzed by fitting a Hill curve using the SigmaPlot 13.0 software to establish the pCa_50_ (pCa at half-maximum force). The cross-sectional area of fibers was calculated from the measurements of their diameters. The maximum Ca^2+^-activated force per cross-sectional area (*F*_max_) is expressed as mN/mm^2^.

### Mitochondrial enzyme activity

The maximal activities of citrate synthase (CS) and cytochrome c oxidase (COX) were determined in whole muscle homogenates. In brief, the whole plantaris muscles were homogenized in ice-cold 100 mM potassium phosphate buffer (100 μl/mg wet wt), and maximal CS and COX activities were measured spectrophotometrically as described previously [40, 41].

### Immunoblotting

Immunoblots were performed as previously described [42] using anti-PGC-1α (ab54481, Abcam), anti-total OXPHOS rodent WB antibody cocktail (ab110413, Abcam), anti-adenosine monophosphate deaminase 1 (AMPD1, NBP2-24509, Novus Biologicals), anti-dystrophin (ab15277, Abcam), anti-glucose-regulated protein (Grp) 78 (ADI-SPA-826, Enzo Life Sciences), anti-Grp94 (ADI-SPA-851, Enzo Life Sciences), anti-inositol-requiring transmembrane kinase/endoribonuclease 1α (IRE1α) (#3294, Cell Signaling), anti-PKR-like endoplasmic reticulum kinase (PERK) (#5683, Cell Signaling), anti-phospho-AMPKα Thr172 (#2531, Cell Signaling), anti-AMPKα (#2532, Cell Signaling), anti-phospho-CaMKII Thr286 (#12716, Cell Signaling), anti-CaMKII (611292, BD Biosciences, San Jose, CA), anti-phospho-ACC Ser79 (#3661, Cell Signaling), anti-ACC (#3662, Cell Signaling), anti-phospho-p38 MAPK (#4511, Cell Signaling), and anti-p38 MAPK (#9212, Cell Signaling).

Muscle pieces were homogenized in ice-cold homogenizing buffer (40 μl/mg wet wt) consisting of (mM) the following: Tris maleate, 10; NaF, 35; NaVO_4_, 1; 1% Triton X 100 (vol/vol); and 1 tablet of protease inhibitor cocktail (Roche) per 50 ml. The protein content was determined using the Bradford assay [43]. Aliquots of the whole muscle homogenates (20 μg) were diluted with Laemmli buffer (mM): urea, 4000; Tris/HCl, 250; SDS, 3.5; 20% glycerol (vol/vol); and 0.0005% bromophenol blue (wt/vol). Proteins were applied to a 4–15% Criterion Stain-Free Gel (BioRad). Gels were imaged (BioRad Stain Free imager), and then proteins were transferred onto the polyvinylidene fluoride membranes and were blocked in 3% (wt/vol) non-fat milk and Tris-buffered saline containing 0.05% (vol/vol) Tween 20, followed by incubation with primary antibody overnight at 4 °C. The membranes were then washed and incubated for 1 h at room temperature with secondary antibody (1:5000, donkey anti-rabbit or donkey anti-mouse, BioRad). Images of the membrane were collected following exposure to chemiluminescence substrate (Millipore) using a charge-coupled device camera attached to ChemiDOC MP (BioRad), and the Image Lab software (BioRad) was used for detection as well as densitometry. The levels of protein expression were normalized to the total proteins from the stain-free image.

### Statistics

Data are presented as mean ± SEM. Data normality was examined with the Shapiro-Wilk test. In *experiment 1*, for normally distributed data (the distribution of the MyHC isoforms, CS activity, COX activity, the expression levels of PGC-1α, NDUFB8, SDHB, UQCRC, MTC01, ATP5, AMPD1, dystrophin, Grp78, Grp94, IRE1α, and PERK, *F*_max_, pCa_50_), one-way ANOVA was used to determine the mean differences among the three groups (CNT, EAM, and EAM+IT group). Fatigue resistance (group × repetitions) and specific force-pCa relationship (group × pCa) were assessed by two-way repeated-measures ANOVA. In *experiment 2*, for normally distributed data (the phosphorylation levels of AMPK, CaMKII, and p38MAPK), one-way ANOVA was used to determine the mean differences between the groups. When these ANOVA tests showed significance, Bonferroni or Tukey post hoc test was performed. If data exhibited a non-normal distribution (the phosphorylation levels of ACC), a Kruskal-Wallis one-way ANOVA was used on ranks. A *P* value less than 0.05 was regarded as statistically significant. A power test was performed assuming changes in physiological measurements after HIIT being 30 ± 20% of the control value. With a power of 0.80 and an alpha of 0.05, this gives a sample size of six. Based on this, we used 6 and 8 animals in each group in *experiments 1* and *2*, respectively, but some analyses were performed with *n* = 5–6 (ER stress proteins). Statistical testing was performed with SigmaPlot (version 13, Systat Software, Inc.).

## Results

### HIIT improves fatigue resistance in the skeletal muscle of EAM mice

In experiment 1, there was no difference in body weight between CNT (*n* = 6) and mice with EAM (*n*= 6) (mean ± SEM 21.4 ± 0.4 g versus 19.1 ± 0.7 g; *P* > 0.05). In contrast, the spleen weight was 2.6-fold higher in the EAM than in the CNT group (mean ± SEM 289 ± 20 mg versus 110 ± 5 mg, *P* < 0.05). The GAS muscle weight was 25% lower in the EAM than in the CNT group (mean ± SEM 70.9 ± 2.2 mg versus 94.2 ± 2.6 mg, *P* < 0.05), and this was not ameliorated by HIIT (mean ± SEM 75.9 ± 3.5 mg, *P* > 0.05).

**Fig. 2:**
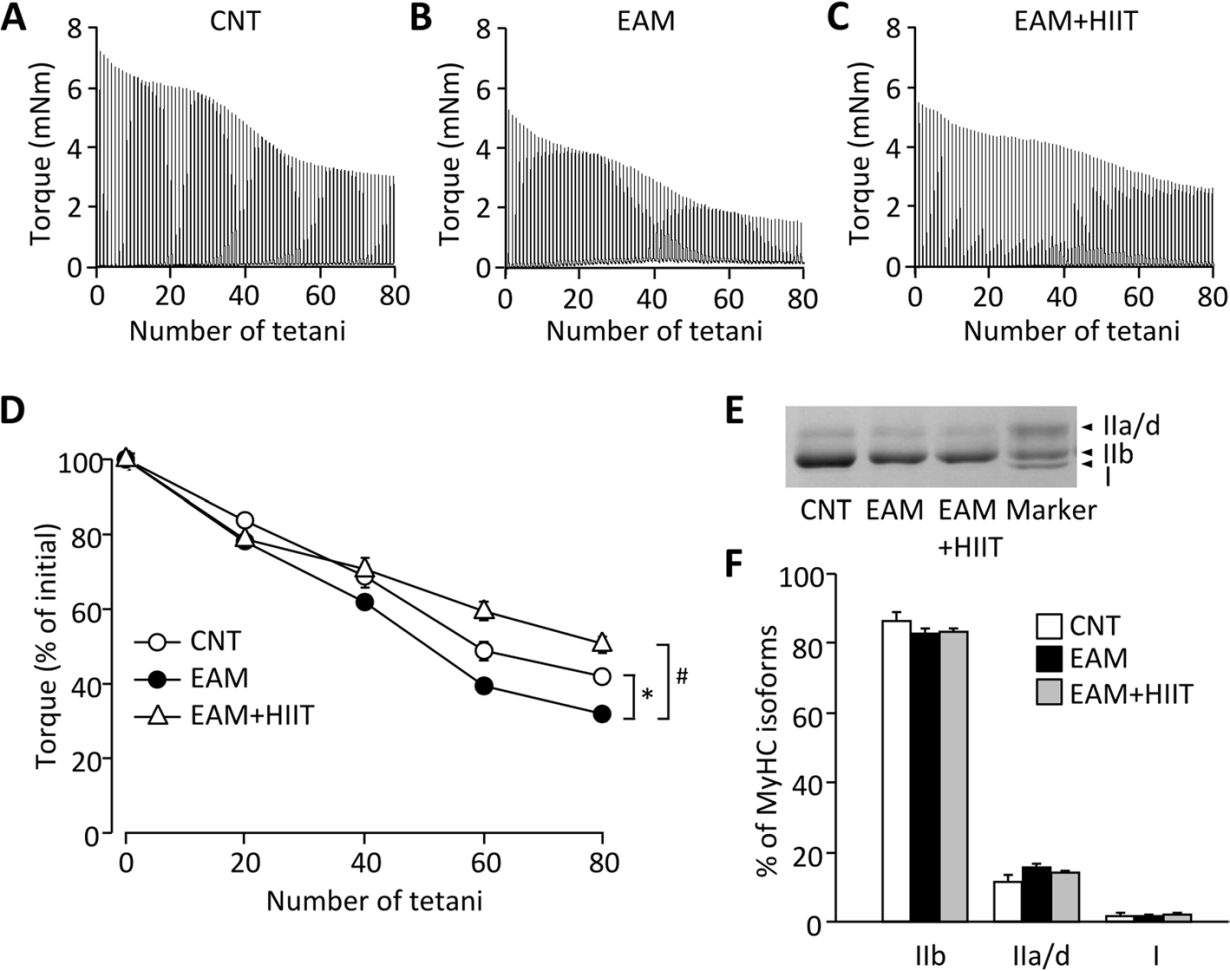
HIIT improves fatigue resistance in skeletal muscle of EAM mice. **A**–**C** Representative torque records during the in vivo fatigue protocol (70 Hz, 350 ms tetani every 3 s) of the plantar flexor muscles from control (CNT) and EAM mice with or without high-intensity interval training (HIIT). **D** Mean (± SEM) relative tetanic torque during fatiguing stimulation. Torque in the first tetanus was set to 100% in each muscle. Two-way repeated-measures ANOVA with Bonferroni post hoc test was performed. *P < 0.05 CNT vs EAM, ^#^*P* < 0.05 EAM vs EAM + HIIT. **E** Blots showing electrophoretically separated myosin heavy chain (MyHC) isoforms in the gastrocnemius muscles in each group. **F** Distribution of MyHC isoforms. Data show the mean and SEM results from 6 muscles per group. One-way ANOVA was performed

Typical torque traces during a HIIT session are shown in Fig. 1B. Note that the torque was decreased much faster in the later sets than in the early sets. Figure 2A–C shows the representative torque records during in vivo fatiguing stimulations of the plantar flexor muscles from CNT and EAM mice with or without HIIT. The EAM muscles were less fatigue resistance than the control muscles (Fig. 2D, *P* < 0.05). Importantly, HIIT significantly improved fatigue resistance in the EAM muscles (*P* < 0.05). The differences in fatigue resistance were not due to any changes in muscle fiber type composition (Fig. 2E, F).

**Fig. 3:**
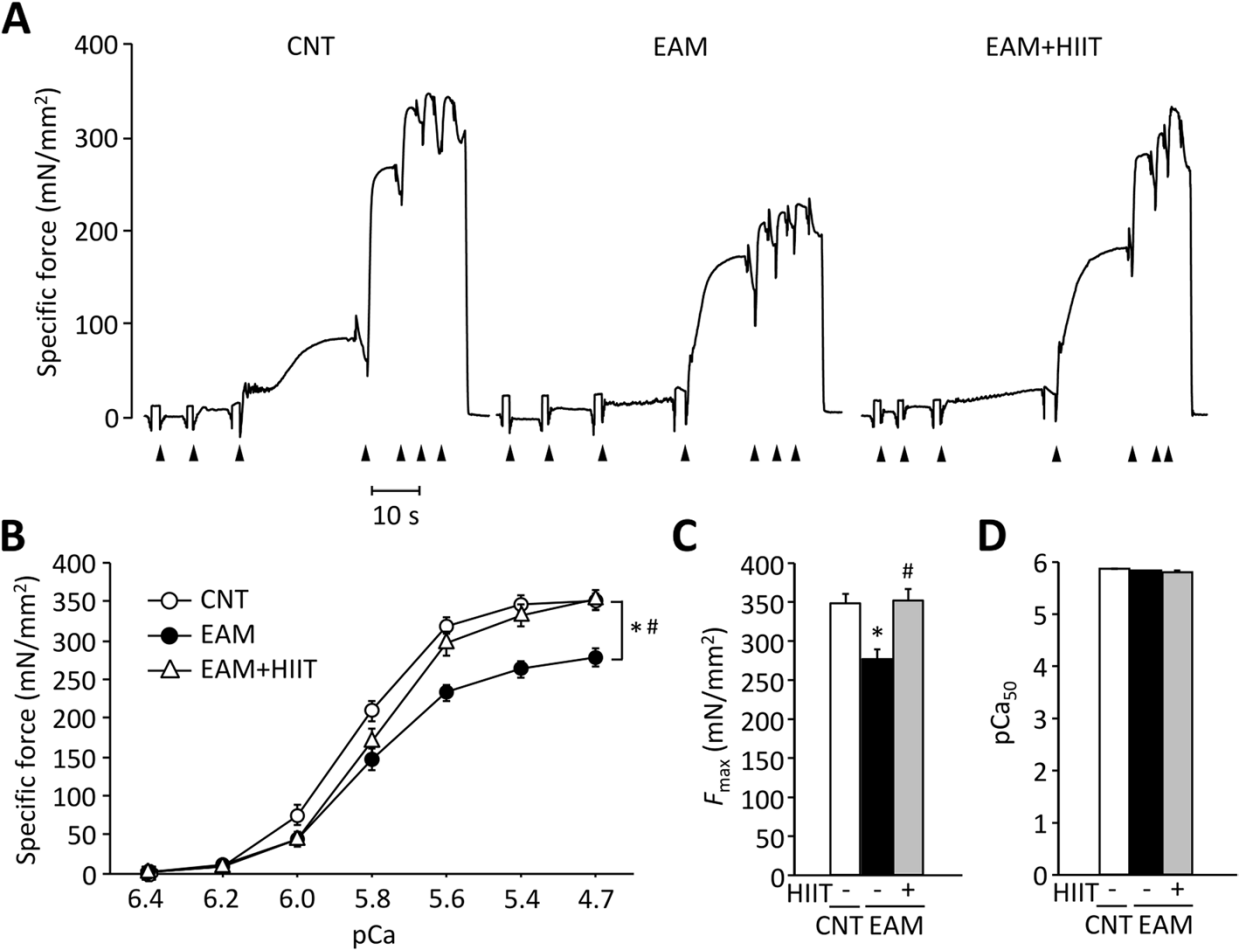
HIIT restores the decrease in myofibrillar force production in the skeletal muscle of EAM mice. **A** Representative original records of in vitro Ca^2+^-activated force in chemically skinned fibers of gastrocnemius muscles from control (CNT) and EAM mice with or without high-intensity interval training (HIIT). Fibers were exposed to solutions with progressively higher free Ca^2+^ concentration: pCa 6.4, 6.2, 6.0, 5.8, 5.6, 5.4, and 4.7. **B** Specific force-pCa relationships. Two-way repeated-measures ANOVA with Tukey post hoc test was performed. **C** Maximum Ca^2+^-activated force per cross-sectional area (*F*_max_). **D** pCa50 (pCa at half-maximum force). Data presented as the mean and SEM from 28 to 31 fibers per group. One-way ANOVA with Bonferroni post hoc test was performed. *P < 0.05 CNT vs EAM, ^#^*P* < 0.05 EAM vs EAM + HIIT

Figure 3A shows the typical traces of Ca^2+^-activated force in skinned fibers from the GAS muscles in each group. The fiber diameter was smaller in the EAM (37.9 ± 0.7 μm [*n* = 31 fibers], *P* < 0.05) and the EAM + HIIT (34.9 ± 1.0 μm [*n* = 31 fibers], *P* < 0.05) groups than in the CNT group (43.6 ± 1.0 μm [*n* = 28 fibers]). Ca^2+^-activated specific force production was lower in skinned fibers from the EAM muscles compared to those from the control muscles (Fig. 3B, *P* < 0.05). Notably, this was restored by HIIT to the control level (*P* < 0.05). The *F*_max_ was 21% lower in the EAM muscle fibers than in the CNT muscle fibers (278 ± 11 mN/mm^2^ versus 349 ± 11 mN/mm^2^, *P* < 0.05), which was recovered by HIIT (353 ± 13 mN/mm^2^, *P* < 0.05) (Fig. 3C). The Ca^2+^ sensitivity (pCa_50_) was similar in the three groups (Fig. 3D, *P* > 0.05).

### HIIT increases mitochondrial respiratory complexes in the skeletal muscle of EAM mice

**Fig. 4:**
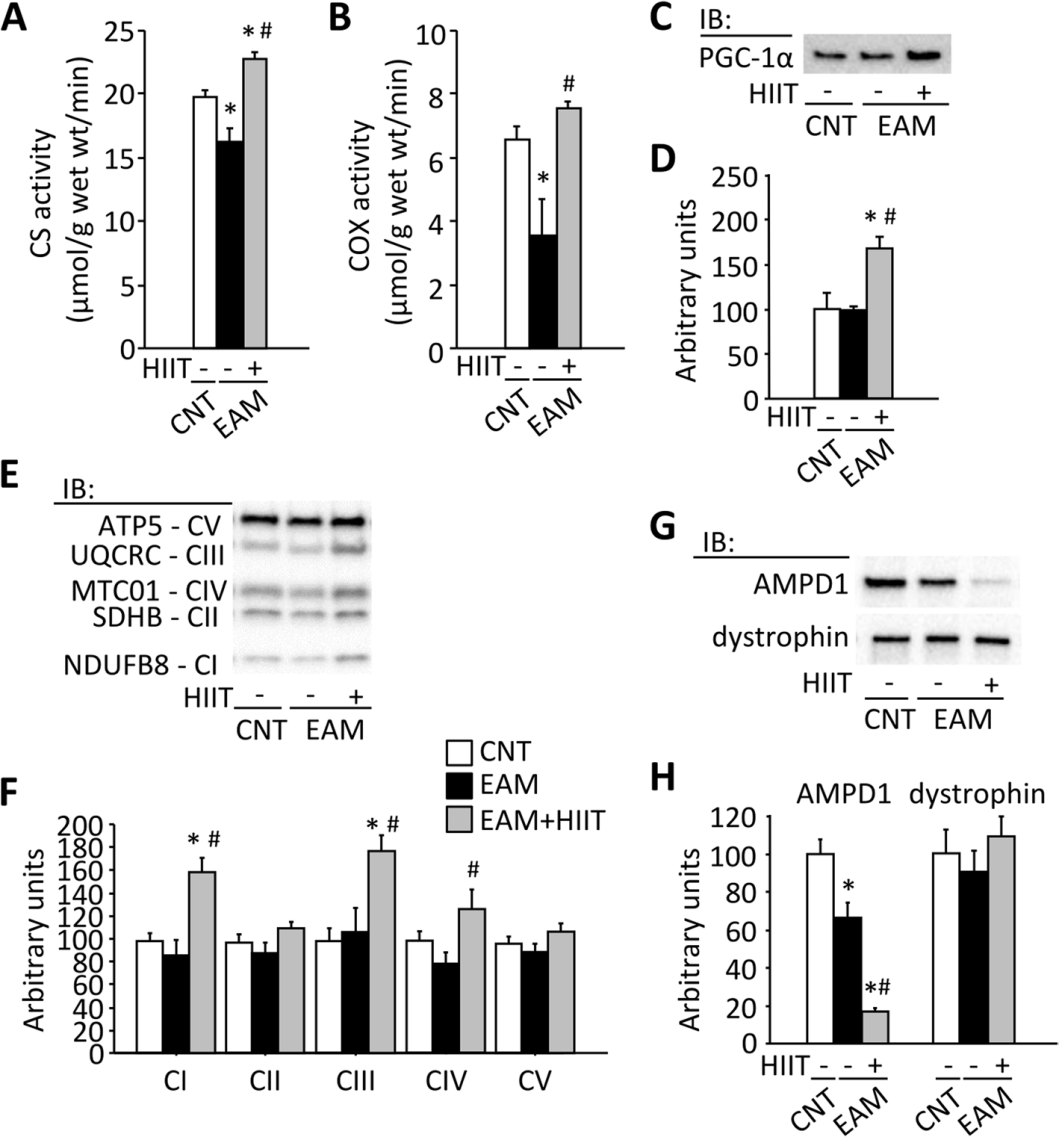
HIIT increases markers of mitochondrial respiration in the skeletal muscle of EAM mice. Citrate synthase (CS) (**A**) and cytochrome c oxidase (COX) (**B**) activities in the plantaris muscles from control (CNT) and EAM mice with or without high-intensity interval training (HIIT). Representative western blots illustrating the levels of peroxisome proliferator-activated receptor γ coactivator-1α (PGC-1α) (**C**); complex I (CI) subunit (NDUFB8), CII subunit (SDHB), CIII subunit (UQCRC), CIV subunit (MTC01), and CV subunit (ATP5) (**E**); and adenosine monophosphate deaminase 1 (AMPD1) and dystrophin (**G**) in the gastrocnemius muscles. The levels of PGC-1α (**D**), CI-V (**F**), AMPD1, and dystrophin (**H**) were normalized to total protein in stain-free images, and the mean in CNT muscles was set to 100%; *n* = 6 muscles per group. One-way ANOVA with Bonferroni post hoc test was performed. *P < 0.05 vs CNT, ^#^*P* < 0.05 vs EAM

Compared to the CNT group, CS and COX activities were lower in the EAM group (Fig. 4A, B, *P* < 0.05). Notably, these EAM-induced deleterious alterations were restored by HIIT (*P* < 0.05). Moreover, HIIT markedly increased the protein expression of PGC-1α and mitochondrial respiratory complexes I, III, and IV in the EAM group (Fig. 4C–F, *P* < 0.05).

Although the precise role in physiology is unsettled, a previous study has proposed that a deficiency of AMPD1, a rate-limiting enzyme involved in the catabolism of AMP to IMP and NH_3_, may contribute to muscle fatigue [44]. The amount of AMPD1 was significantly lower in the EAM group than in the CNT group (Fig. 4G, H, *P* < 0.05). In contrast, HIIT further reduced the amount of AMPD1 in the EAM muscles (*P* < 0.05). Additionally, an acquired reduction in dystrophin has been found in patients with SAMs [45] and a class I major histocompatibility complex (MHC)-transgenic mouse model of SAMs [46]. However, there was no difference in the amount of dystrophin between the groups (Fig. 4G, H, *P* > 0.05).

### HIIT alleviates ER stress in the skeletal muscle of EAM mice

**Fig. 5:**
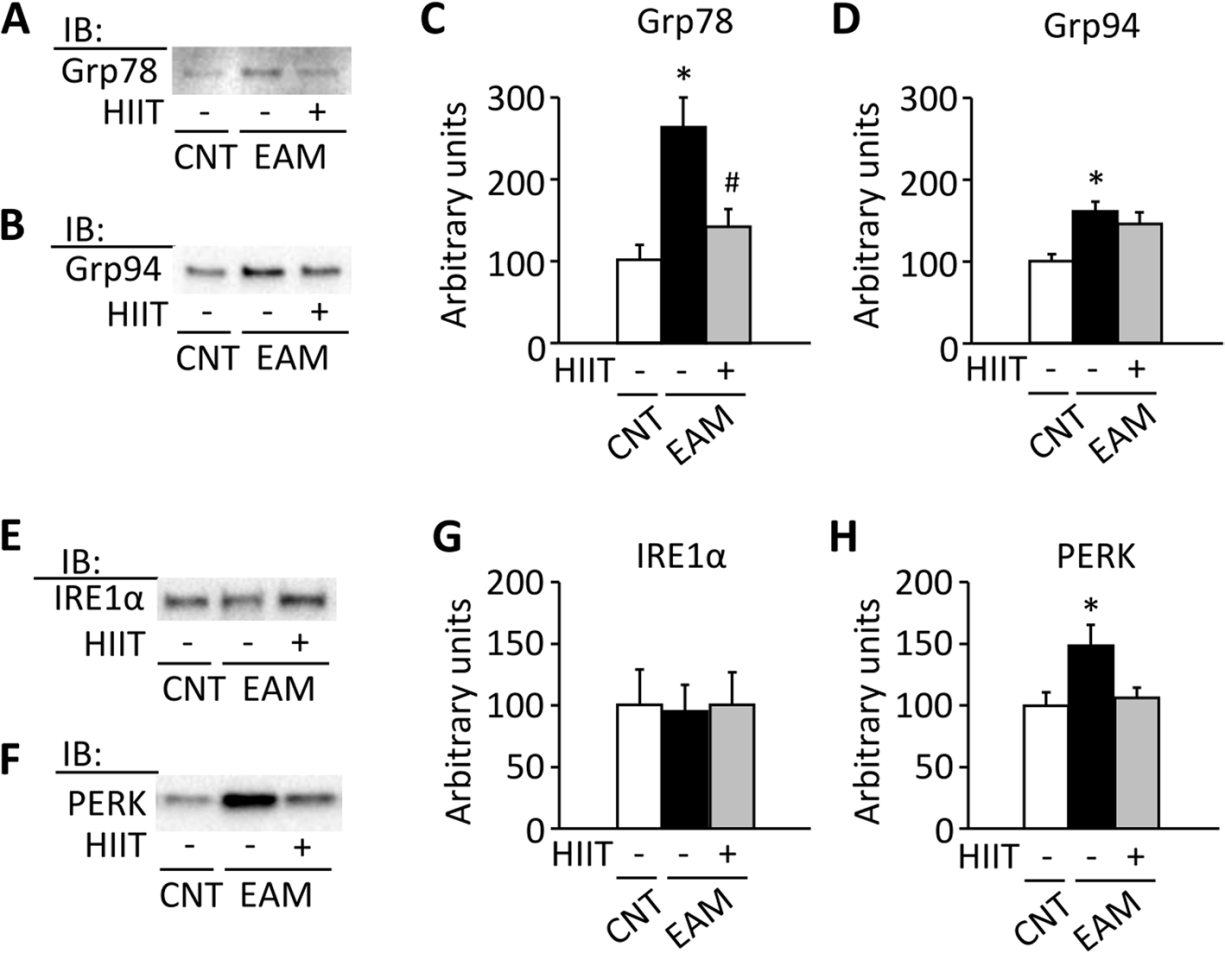
HIIT alleviates the upregulation of ER stress proteins in the gastrocnemius muscle of EAM mice. Representative western blots illustrating the levels of glucose-regulated protein (Grp) 78 (**A**), Grp94 (**B**), inositol-requiring transmembrane kinase endoribonuclease-1α (IRE-1α) (**E**), and PKR-like ER kinase (PERK) (**F**) of the gastrocnemius muscles in control (CNT) and EAM mice with or without high-intensity interval training (HIIT). Bars showing the mean and SEM levels of these proteins (**C**, **D**, **G**, **H**). Data were normalized to the total proteins from the stain-free image, and the mean in CNT muscles was set to 100%; *n* = 5–6 muscles per group. One-way ANOVA with Bonferroni post hoc test was performed. *P < 0.05 vs CNT, ^#^*P* < 0.05 vs EAM

Previous studies suggest that the ER stress pathways are chronically activated and may play an etiological role in SAM [9]. Accordingly, the GAS muscles of EAM mice showed significantly increased expression of the unfolded protein response proteins Grp78, Grp94, and PERK, but not IRE-1α (Fig. 5A–H, *P* < 0.05). Notably, HIIT attenuated the increased expressions of these ER stress-related proteins.

### The phosphorylation levels of signaling proteins are increased after a single bout of HIIT

**Fig. 6:**
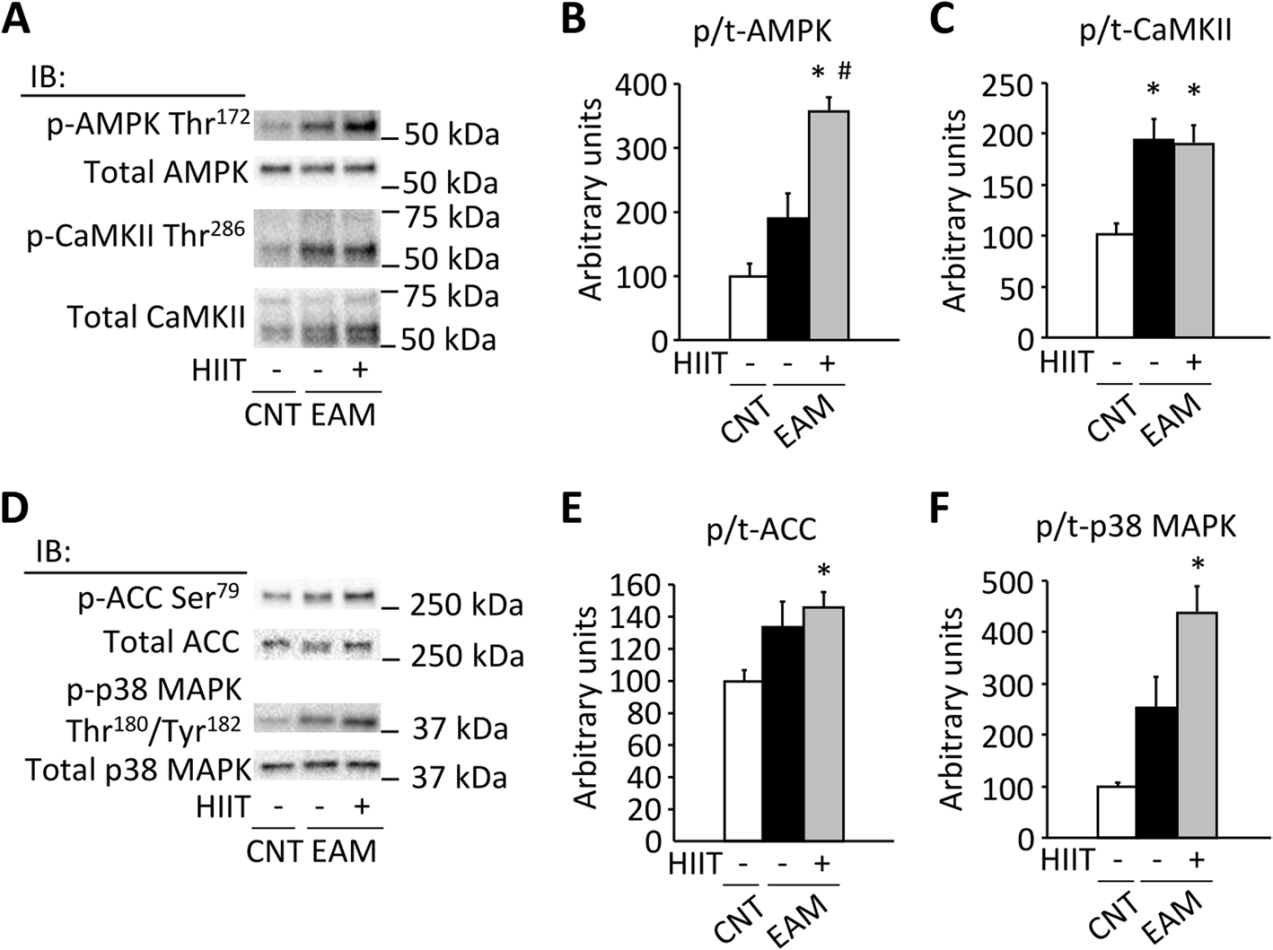
The phosphorylation levels of signaling proteins are increased after a single bout of HIIT. Representative western blots of the total and phosphorylated AMPK Thr172, CaMKII Thr286 (**A**), ACC Ser79, and p38 MAPK Thr180/Tyr182 (**D**) of the gastrocnemius muscles in control (CNT) and EAM mice with or without one bout of high-intensity interval training (HIIT; Exp 2). Bars showing the mean and SEM of the phosphorylation levels of AMPK Thr172 (**B**), CaMKII Thr286 (**C**), ACC Ser79 (**E**), and p38 MAPK Thr180/Tyr182 (**F**) relative to total protein content. Data expressed relative to the mean in the CNT muscles, which was set to 100%; *n* = 10 muscles per group. One-way ANOVA with Tukey post hoc test was performed, except for the phosphorylation level of ACC where a Kruskal-Wallis one-way ANOVA was used on ranks. *P < 0.05 vs CNT, ^#^*P* < 0.05 vs EAM

In experiment 2, the body weight was slightly higher in mice with EAM (*n* = 8) than in mice with CNT (*n* = 8) (mean ± SEM 19.4 ± 0.3 g versus 18.0 ± 0.3 g; *P* < 0.05). The spleen weight was 5-fold higher in the EAM than in the CNT group (mean ± SEM 432 ± 11 mg versus 87 ± 4 mg, *P* < 0.05). The phosphorylation levels of AMPK Thr172, ACC Ser79, and p38 MAPK Thr180/Tyr182 did not differ between the CNT and the EAM groups, while the phosphorylation levels of these molecules were increased immediately after one HIIT session compared to the CNT group (Fig. 6A, B, D, E, F, *P* < 0.05). On the other hand, CaMKII Thr286 phosphorylation was higher in the EAM group than in the CNT group (Fig. 6A, C, *P* < 0.05), which was not affected by an HIIT session.

## Discussion

In accordance with our hypothesis, we show a reduced fatigue resistance during in vivo fatiguing stimulation in the skeletal muscle of EAM mice, an animal model for acute SAMs [30]. The decreased fatigue resistance was accompanied by the increased expression of ER stress-related proteins and reduced activities of mitochondrial oxidative enzymes. Importantly, these deleterious events were restored by HIIT starting 24 h after the last immunization where muscle function is impaired.

Theoretically, the fatigue resistance of muscle fiber depends on the fiber type, which is defined by the MyHC isoform. However, the impaired endurance performance was not accompanied by an alteration in MyHC isoforms in the skeletal muscle of EAM mice, an animal model for acute SAMs. In line with this, untreated newly diagnosed patients with PM/DM had a similar fiber type composition to healthy individuals, although patients with chronic PM/DM display fewer slow-twitch type I fibers [47]. Thus, alterations in muscle fiber types towards more fatigable isoforms likely contribute to the reduced fatigue resistance at chronic stages, but not at disease onset, of SAMs. On the other hand, previous studies suggest a low mitochondrial respiratory capacity as an important mechanism contributing to the impaired endurance performance in patients with SAMs [2, 5, 6]. In agreement, our data show that the reduced fatigue resistance is accompanied by decreased activities of CS and COX in the skeletal muscle of mice with EAM.

The mechanisms underlying the impaired mitochondrial function in SAMs remain uncertain, although non-immune-mediated pathways are thought to be involved. Indeed, despite the recommended treatment with conventional immunosuppressive agents, few SAM patients regain full muscle endurance performance [2]. In this regard, there is growing evidence to suggest that ER stress pathways are chronically activated in SAMs [9] and are linked to mitochondrial dysfunction [10, 48]. Recently, Thoma et al. [11] have shown that the ER stress inducer, tunicamycin, promotes mitochondrial dysfunction in a human skeletal muscle cell line. Moreover, it has been demonstrated that PERK, a key ER stress sensor of the unfolded protein response, resides in MAMs and plays a critical role in mitochondrial dysfunction [12]. Accordingly, our findings of the decreased activities of mitochondrial oxidative enzymes in combination with the increased ER stress proteins Grp78, Grp94, and PERK suggested that sustained ER stress underlies mitochondrial dysfunction in the skeletal muscle of EAM mice.

The improvement of fatigue resistance by HIIT in EAM can be explained by increased muscle aerobic capacity as judged by the upregulation of mitochondrial respiratory complexes (I, III, and IV) and increased activities of CS and COX. The increased CS activity and fatigue resistance have also been reported in patients with PM/DM who performed 12 weeks of endurance training [2, 18]. Notably, one HIIT session in the skeletal muscle of EAM mice was followed by phosphorylation of AMPK, ACC, and p38 MAPK, which was associated with increased PGC-1α protein expression after 4 weeks of HIIT. Importantly, we recently have demonstrated that the same protocol done with normal mice also improves resistance to fatigue accompanied by similar molecular changes in PGC-1a and mitochondrial function [23], indicating that the experimental model is standardized and validated in healthy animals. Accordingly, these data indicate that the PGC-1α-dependent augmentation of mitochondrial oxidative capacity can be effectively induced by exercise training even under inflammatory conditions such as SAMs.

In addition to mitochondrial biogenesis, HIIT may improve muscle aerobic capacity by ameliorating mitochondrial dysfunction due to ER stress in EAM mice. Indeed, our data show that HIIT inhibited the increased amount of ER stress proteins Grp78, Grp94, and PERK in the skeletal muscle of EAM mice. This is in line with a previous study from our lab where EAM-induced upregulation of ER stress proteins, including Grp78 and Grp94, was attenuated by 4 weeks of high-intensity eccentric contraction training in EAM mice [31]. Taken together, our findings promote exercise as an important non-pharmacological approach for relieving ER stress and improving mitochondrial function. Although the mechanisms underlying this beneficial effect of exercise remain unresolved, previous studies suggest that AMPK functions as a suppressor of ER stress [29, 49].

The skeletal muscle of patients with SAMs [50, 51] and a class I MHC-transgenic mouse model of SAMs [52] exhibit a reduction of AMPD1, which catalyzes the deamination of AMP to IMP and plays an important role in the purine nucleotide cycle. It has been proposed that AMPD1 deficiency is responsible for muscle weakness in a class I MHC-transgenic mouse [52]. Although we also observed a reduction in AMPD1 content in the skeletal muscle of EAM mice, HIIT-induced increase in fatigue resistance was accompanied by decreased rather than increased amount of AMPD1 in those muscles. Several studies of human subjects have reported variable results with some studies suggesting the mutation of the AMPD1 gene may cause easy fatigability while others indicate individuals with this inherited defect are completely asymptomatic [53]. Cheng et al. [54] have revealed using AMPD1 knockout mice that AMPD1 deficiency results in no abnormality in muscle performance in both sprint and endurance exercise protocols. Thus, these data suggest that an AMPD1 deficiency may not be involved in the mechanism underlying reduced fatigue resistance in patients with SAMs.

### Study limitations

There is much debate about which animal model better mimics different aspects of pathology in SAMs. It would be intriguing for a future study to perform the same experiments using an alternative model. For example, a class I MHC-transgenic mouse model of SAMs has recently been shown to exhibit muscle weakness in combination with an acquired reduction in dystrophin [46] in line with what is seen in many patients [45]. This acquired reduction in dystrophin could possibly lead to increased muscle damage from eccentric contractions or HIIT, in contrast to the EAM model used here, which does not have a reduction in dystrophin.

## Conclusions

We here show reduced fatigue resistance in the skeletal muscle of a mouse model of acute SAM. This functional defect was due to decreased mitochondria oxidative capacity, which was at least in part caused by activation of ER stress-dependent pathway. HIIT-mimicking electrical stimulation reversed these alterations and markedly improved fatigue resistance without any signs of deleterious effects on the skeletal muscle. Thus, our findings highlight the clinical importance of HIIT as a safe and effective way to treat increased muscle fatiguability in patients with SAMs.

## Data Availability

The dataset supporting the conclusions of this article is included within the article.
